# Interleukin 8 activity influences the efficacy of adenoviral oncolytic immunotherapy in cancer patients

**DOI:** 10.18632/oncotarget.23967

**Published:** 2018-01-05

**Authors:** Kristian Taipale, Siri Tähtinen, Riikka Havunen, Anniina Koski, Ilkka Liikanen, Päivi Pakarinen, Riitta Koivisto-Korander, Matti Kankainen, Timo Joensuu, Anna Kanerva, Akseli Hemminki

**Affiliations:** ^1^ Cancer Gene Therapy Group, University of Helsinki, Faculty of Medicine, Helsinki, Finland; ^2^ Department of Neurosurgery, HUCH, Helsinki, Finland; ^3^ Department of Obstetrics and Gynecology, HUCH, Helsinki, Finland; ^4^ Institute for Molecular Medicine Finland (FIMM), University of Helsinki, Helsinki, Finland; ^5^ Docrates Cancer Center, Helsinki, Finland; ^6^ TILT Biotherapeutics Ltd., Helsinki, Finland; ^7^ Helsinki University Hospital Comprehensive Cancer Center, Helsinki, Finland

**Keywords:** oncolytic adenovirus, neutrophils, anti-tumor immunity, cytokines, immunomodulation

## Abstract

After the landmark approval of T-VEC, oncolytic viruses are finding their way to the clinics. However, response rates have still room for improvement, and unfortunately there are currently no available markers to predict responses for oncolytic immunotherapy. Interleukin 8 (IL-8) production is upregulated in many cancers and it also connects to several pathways that have been shown to impair the efficacy of adenoviral immunotherapy. We studied the role of IL-8 in 103 cancer patients treated with oncolytic adenoviruses. We found high baseline serum IL-8 concentration to be independently associated with poor prognosis (p<0.001). Further, normal baseline IL-8 was associated with improved prognostic potential of calculation of the neutrophil-to-lymphocyte ratio (p<0.001). Interestingly, a decrease in IL-8 concentration after treatment with oncolytic adenovirus predicted better overall survival (p<0.001) and higher response rate, although this difference was not significant (p=0.066). We studied the combination of adenovirus and IL-8 neutralizing antibody *ex vivo* in single cell suspensions and in co-cultures of tumor-associated CD15+ neutrophils and CD3+ tumor-infiltrating lymphocytes derived from fresh patient tumor samples. These results indicate a role for IL-8 as a biomarker in oncolytic virotherapy, but additionally provide a rationale for targeting IL-8 to improve treatment efficacy. In conclusion, curtailing the activity of IL-8 systemically or locally in the tumor microenvironment could improve anti-tumor immune responses resulting in enhanced efficacy of adenoviral immunotherapy of cancer.

## INTRODUCTION

After years of development, the first oncolytic viruses are currently entering the clinics as cancer therapeutics with products approved in both the East and West [1–3]. Despite recent discoveries relating to the mechanisms of action and factors that influence the efficacy of oncolytic viruses [4–6], there is still a critical need to identify pathways that determine the response to virotherapy and survival benefits thereafter. In this regard, analysis of patient data is of key importance since immunological effects of human-specific agents may significantly differ between humans and experimental animals. In clinical trials, oncolytic viruses have demonstrated a favorable safety profile and promising anti-tumor efficacy [7]. However, there is still room for improvement with regard to the frequency of anti-tumor responses, especially in patients with significant metastasis burden. Identification and characterization of pathways associated with the activity of oncolytic viruses could reveal potential targets for improving the efficacy of virotherapy. Moreover, understanding the key molecular mechanisms would help selection of the right patients for therapy.

Interleukin 8 (IL-8) was originally discovered as a neutrophil chemoattractant isolated from human monocytes in the 1990’s [8]. Since then, activated IL-8 signaling has been identified in different cancers with one of its many effects relating to regulation of angiogenesis [9]. In tumors, IL-8 has also been proposed to have a role in promotion of survival, cell proliferation, chemoresistance and metastasis formation [10, 11]. There have been several attempts to therapeutically target the IL-8 pathway in the context of cancer and other diseases, and some approaches have demonstrated promising efficacy [12–15]. However, to date no therapies targeting IL-8 have been approved for clinical use.

A number of different viruses have been shown to promote IL-8 production and it is a key mediator of neutrophil dependent pathogenesis in many viral infections [16–18]. Some adenovirus serotypes, notably serotypes 7 and 19 [19, 20], also upregulate IL-8 and recruit neutrophils to the infection site. Interestingly, considering the use of viruses in cancer immunotherapy, systemic neutrophil expansion has been linked to metastasis formation in murine tumor models [21]. In contrast to the wild-type viruses, at least one oncolytic adenovirus has also been recently shown to decrease IL-8 signaling in preclinical tumor models [22].

To our knowledge, there are no previous human data on the effects of IL-8 in the context of oncolytic viruses, excluding limited data from the phase II trial of oncolytic reovirus [23]. We have previously demonstrated that high levels of pre-existing activity of innate immune system results in lower survival of patients receiving oncolytic adenovirus [24], and – conversely - that patients with low baseline immune activation are more likely to benefit from oncolytic immunotherapy. This probably relates to the unparalleled ability of oncolytic viruses to turn immunologically cold tumors into hot inflamed tumors [2], which may underlie an important part of their mechanism of action. In tumors already inflamed, this aspect of the therapy probably adds little (although oncolytic cell lysis could still result in anti-tumor effects). Additionally, low pre-treatment levels of HMGB1, which is associated with immunogenic cell death but also mediates chronic inflammation under a steady state, have been linked to better survival and therapeutic efficacy [25, 26]. This background provides a strong rationale for investigation of the role of IL-8 in immunotherapy with oncolytic viruses.

The aim of this study was to examine how IL-8 activity shapes the responses to treatment with oncolytic adenovirus. We analyzed baseline serum IL-8 in 103 patients treated with oncolytic adenovirus and measured post-treatment changes in IL-8. We then compared these findings to available survival and response data. In additional analyses, we compared serum IL-8 levels to tumor type and tumor load. We also assessed how different treatment characteristics affect IL-8 changes. Due to the close connection between IL-8 and neutrophils, their associations were investigated in peripheral blood. To evaluate the role of tumor-derived IL-8 on survival, expression levels of IL-8 and its receptors CXCR1 and CXCR2 were quantified by RNA microarrays from pre- and post-treatment tumor samples from oncolytic virus treated patients. Finally, we analyzed the anti-tumor and immunostimulatory activity of a combination of IL-8 blockade and oncolytic adenovirus in human ovarian tumor samples obtained fresh from the operating room.

## RESULTS

### Normal baseline IL-8 before treatment with oncolytic adenovirus correlates with longer overall survival

**Figure 1:**
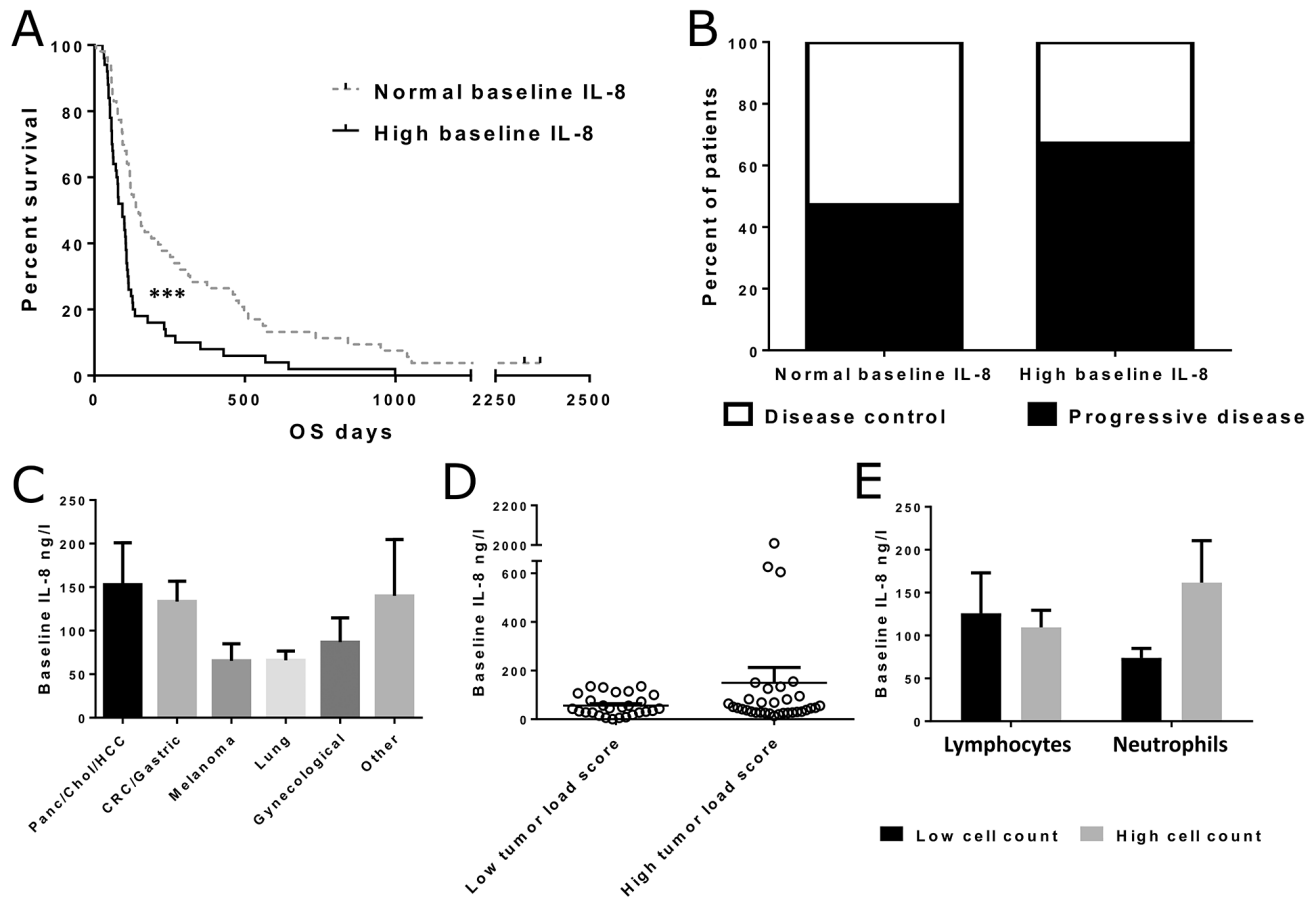
Overall survival and treatment responses in patients with normal and high pre-treatment IL-8 levels in blood and IL-8 concentrations in different tumor groups Patients were grouped based on the laboratory reference value for normal IL-8 (<62 ng/l). Asterisks indicate statistical significance: ^***^ p<0.001, ^**^ p<0.01 and ^*^ p<0.05. **(Panel A)** Overall survival was significantly longer in patients with normal IL-8 before treatment (n=53, median OS 147 days) when compared with high IL-8 patients (n=50, median OS 93 days, p<0.001). **(Panel B)** Imaging response information was available for 59 patients, with baseline IL-8 measurements (normal IL-8 n=38, high IL-8 n=21). No significant difference in disease control rate was observed between the IL-8 groups (Fisher’s exact test p=0.182). **(Panel C)** Mean pre-treatment IL-8 concentrations in patients with different tumor types. The differences were not considered significant. Error bars are shown as mean + SEM in all panels. **(Panel D)** Scatter plot presenting IL-8 levels in patients with high and low tumor load scores. Vertical line indicates sample mean. The difference was not significant (p=0.187). **(Panel E)** Mean IL-8 concentrations in patients with high and low lymphocyte/neutrophil counts. Patients were grouped based on median lymphocyte/neutrophil count. Difference between high and low neutrophil count patients was not significant (p=0.085).

Baseline IL-8 levels were measured from pre-treatment peripheral blood samples of 103 patients (Supplementary Table 1). Patients were divided into high and low baseline groups based on the “normal” laboratory reference range for serum IL-8 (“normal” is below 62 ng/l). Survival between high and low baseline groups was compared using the Kaplan-Meier method. We found significant differences in the survival of high and low baseline IL-8 patients (p<0.001) (Figure 1A). We also measured other inflammatory cytokines, including IL-6, IL-10, TNFα and GM-CSF, in baseline serum samples, but found no differences in overall survival when using reference values or even sample median as cutoff (Supplementary Figure 1A-1D). Despite a clear difference in survival, there was only a non-significant trend between baseline IL-8 and treatment response, although the number of patients imaged and the nowadays well appreciated phenomenon of pseudoprogression [27] could have impacted the p-value. The proportions of patients displaying disease control were 53% and 33% in IL-8 low and high groups, respectively (p=0.182) (Figure 1B).

### IL-8 levels in patients with different tumor types, tumor load and pre-treatment leucocyte counts

Different tumor types had slightly diverging average serum IL-8 levels (Figure 1C), but the differences were not considered statistically significant. When patients were grouped based on the calculated tumor load score, which reflected the overall tumor burden of the patient, average serum IL-8 concentrations seemed to be slightly higher, although not significantly, in patients with high tumor load score (Figure 1D). This difference was mainly due to three outlier patients with exceptionally high IL-8 levels.

Since IL-8 is known to function as a chemoattractant for immune cells, and especially neutrophils, we analyzed the pre-treatment cell counts of two major leucocyte subsets, neutrophils and lymphocytes, in 86 patients for whom also IL-8 data was available. We then grouped the patients into high and low lymphocyte and neutrophil count groups, and compared baseline IL-8 levels between these groups (Figure 1E). Difference in IL-8 levels was larger between the low and high neutrophil groups in comparison to the difference between low and high lymphocyte groups, although the neutrophil groups did not differ significantly (p=0.085).

### Post-treatment decrease in IL-8 independently predicts improved overall survival

**Figure 2:**
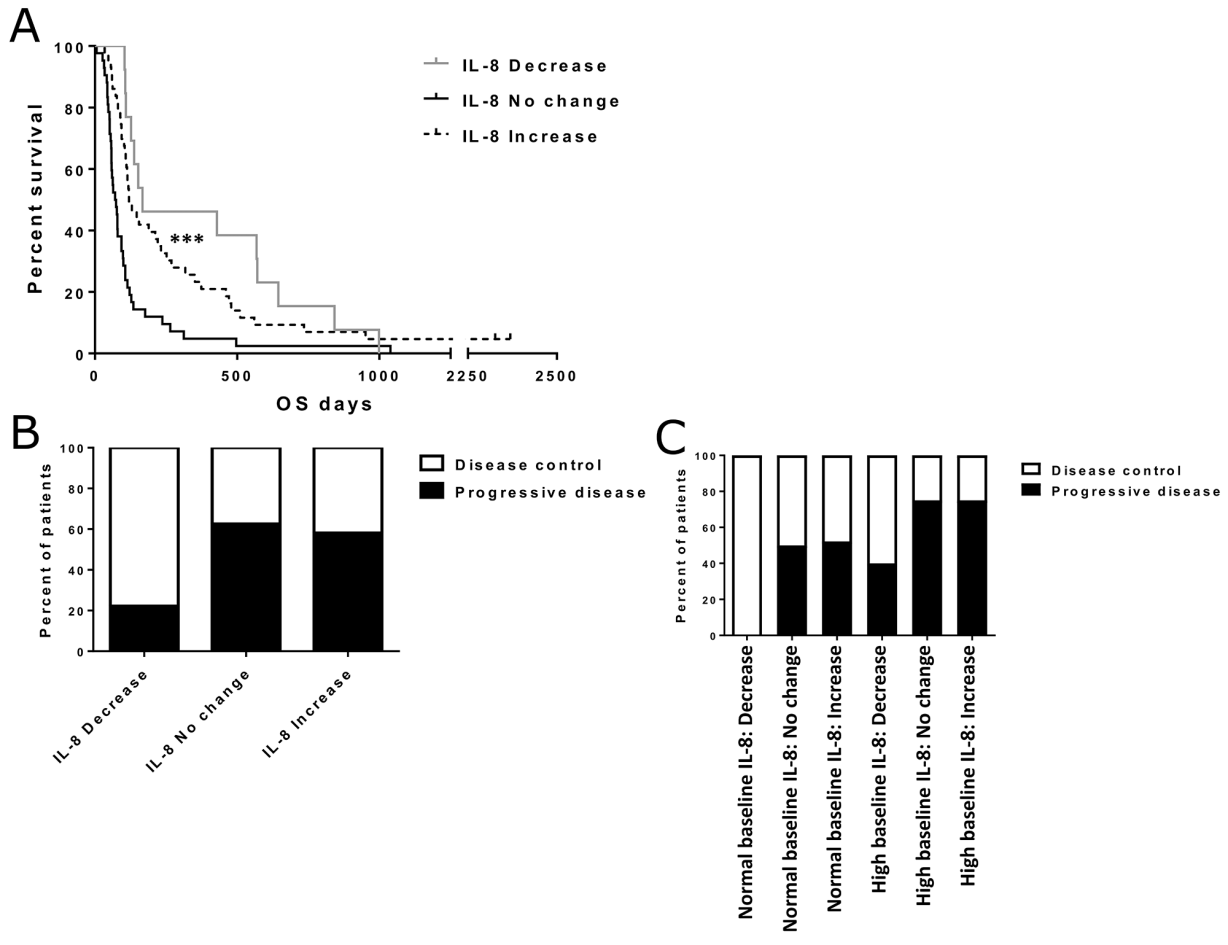
Effect of IL-8 change status on overall survival and treatment responses Asterisks indicate statistical significance: ^***^ p<0.001, ^**^ p<0.01 and ^*^ p<0.05. **(Panel A)** Overall survival in different IL-8 change groups. Median survivals were 167, 71 and 120 days for decrease, increase and no change groups, respectively (p<0.001). **(Panel B)** Disease control rates for patients with IL-8 decrease (n=9), increase (n=31) and no change (n=16). Fisher’s exact test p=0.066 (after pooling no change and increase groups). **(Panel C)** Separate disease control rates in IL-8 change groups for patients with normal or high baseline IL-8 (normal IL-8: decrease n=4, no change n=8, increase n=23. high IL-8: decrease n=5, no change n=8, increase n=8). The differences in disease control rates were not considered significant (Fisher’s exact test p=0.177).

We measured changes in IL-8 levels, following each patient’s first treatment with oncolytic adenovirus. Patients were categorized into increase, decrease and no-change groups based on post-treatment IL-8 levels (Supplementary Figure 2). The minimal requirements for increase and decrease were a 100% growth and a 50% decline from baseline IL-8 levels during adenoviral immunotherapy, respectively. We analyzed survival between these three groups and found patients with post-treatment IL-8 decrease to have significantly longer overall survival (p<0.001) (Figure 2A).

Imaging responses after treatment were also compared between IL-8 change groups (Figure 2B). Interestingly, this comparison revealed an almost two-fold difference in disease control rate between IL-8 decrease and the other two groups. However, partly due to the small sample size, the difference did not reach statistical significance despite pooling of the increase and no-change groups (Fisher’s exact test p=0.066). In additional analyses, when both baseline IL-8 status and IL-8 change were taken into account, decrease in IL-8 seemed to correlate with a higher disease control rate. The effect was similar in both high and low baseline groups (Figure 2C), but the differences were not considered significant.

To further validate the survival effect of both baseline IL-8 and IL-8 change, we constructed a multivariate proportional hazards model with clinical variables of the patients (Supplementary Table 2). In this analysis, normal baseline IL-8 and post-treatment IL-8 decrease were associated with significantly lower hazard ratios for tumor related mortality (HR 0.502, p=0.010 and HR 0.270, p=0.001, respectively), which supports their role as independent prognostic factors for adenoviral immunotherapy. Impressively, IL-8 decrease was the strongest prognostic factor in this analysis, even stronger than the general condition (WHO classification) or tumor type of the patients.

### Treatment characteristics do not explain the observed changes in IL-8 levels

To investigate whether the characteristics of the treatment influence the post-treatment IL-8 change, we correlated IL-8 changes with treatment virus type (capsid), the arming device (transgene) or concomitant “virus sensitizing” treatment (Supplementary Figure 3). In these comparisons, we observed no significant differences between patients who received virus coding for no transgenes or coding the immunostimulatory granulocyte-macrophage colony-stimulating factor (GM-CSF) (Supplementary Figure 3A) or virus with Ad5 capsid [28] or chimeric Ad5/3 capsid [29] (Supplementary Figure 3B). Additionally, IL-8 changes were not impacted by concomitant low-dose cyclophosphamide which was used to reduce regulatory T-cells [30] (Supplementary Figure 3C).

### IL-8 and IL-8 receptor RNA expression in tumor samples

**Figure 3:**
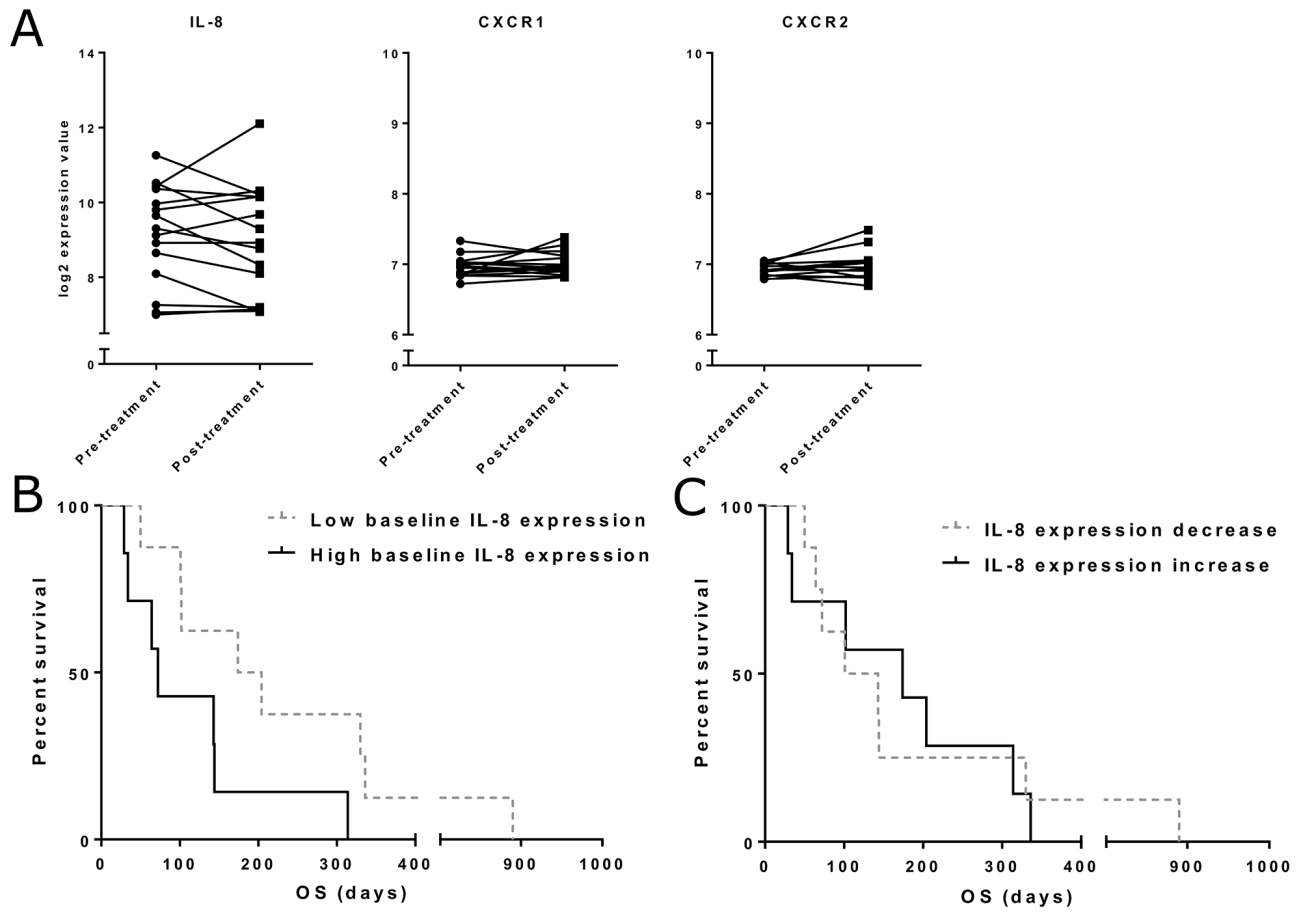
Tumor IL-8 and IL-8 receptor mRNA expression **(Panel A)** mRNA expression for IL-8 and its receptors CXC chemokine receptor 1 (CXCR1) and 2 (CXCR2) was quantified from pre- and post-treatment tumor samples. Expression is presented as log2-transformed values. **(Panel B)** Overall survival in different IL-8 expression groups. Patients were grouped based on the pre-treatment IL-8 expression level. Median OS was 174 days in the low expression group and 72 days in the high expression group (n=15, p=0.058). **(Panel C)** IL-8 expression change was determined as the difference between pre- and post-treatment expression values. No significant difference in survival between decrease and increase groups was found.

To quantify IL-8 at the tumor site, we measured RNA expression from pre- and post-treatment tumor or ascites/pleural fluid samples from an additional cohort of 15 patients treated with oncolytic adenoviruses (Supplementary Table 3). Together with IL-8, we analyzed expression of the two IL-8 receptors CXCR1 and CXCR2 (Figure 3A). Variation in pre-treatment expression levels and pre-post changes was remarkably larger for IL-8 compared to its receptors, and thus we focused on IL-8 in subsequent analyses.

For survival analysis, patients were grouped based on the baseline tumor-level expression of IL-8 mRNA and pre-post treatment change in expression (Figure 3B–3C). Change in IL-8 expression was not correlated with overall survival, but as seen for IL-8 analysis in blood, we observed an interesting trend for longer survival in patients with low pre-treatment IL-8 expression in tumors, although the difference did not reach statistical significance (p=0.058) in this small patient cohort.

### Baseline IL-8 status improves the prognostic value of anti-tumor T cell ELISPOT activity and neutrophil-to-lymphocyte ratio

**Figure 4:**
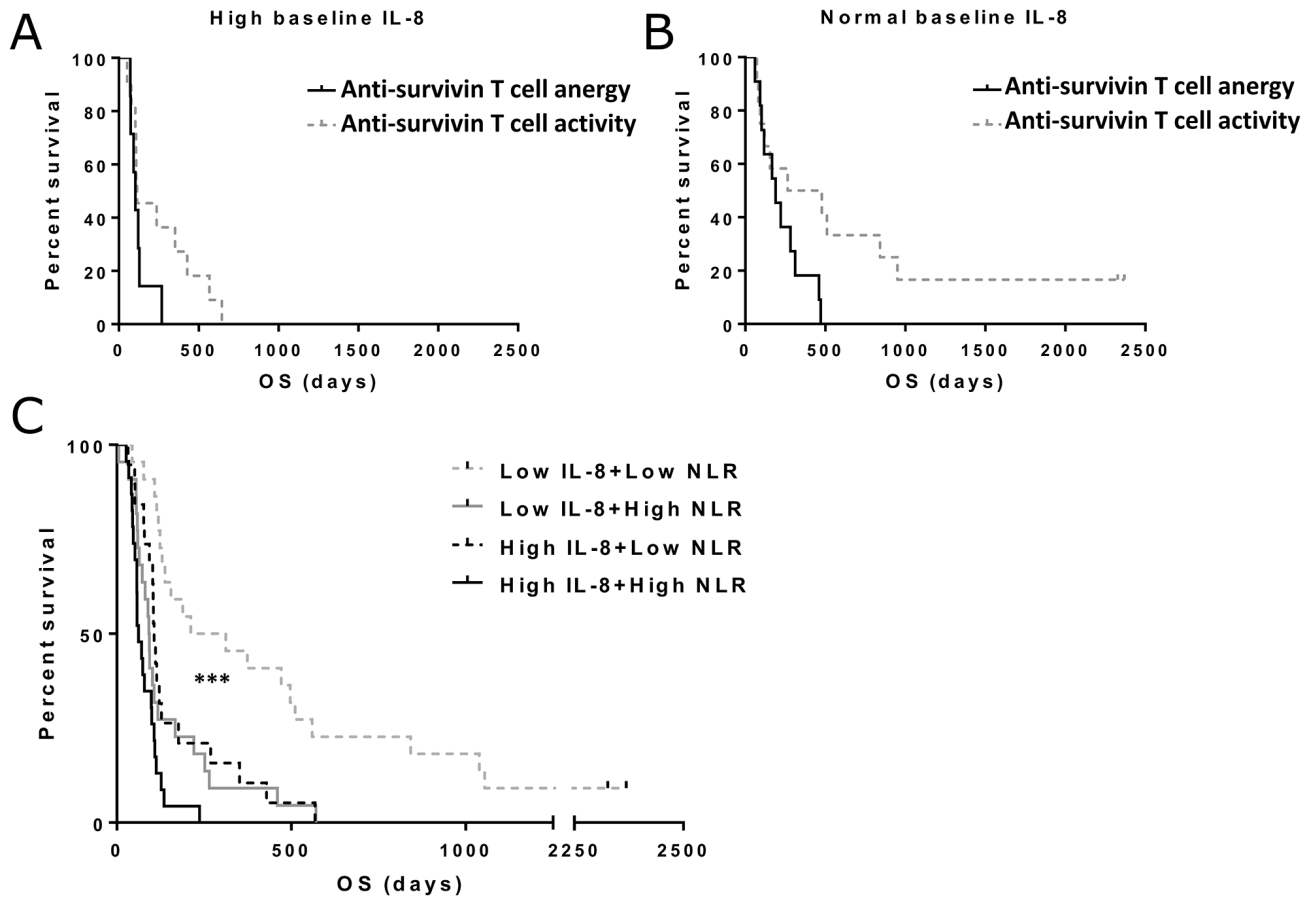
Overall survival in patients with different baseline T cell activity status, neutrophil-to-lymphocyte ratio (NLR) and IL-8 levels Patients with increase or decrease in post-treatment anti-survivin were included in the T cell activity group, while patients with no change in the ELISPOT were assigned to anergy group. Asterisks indicate statistical significance: ^***^ p<0.001, ^**^ p<0.01 and ^*^ p<0.05. **(Panel A)** Median overall survivals in high baseline IL-8 patients were 104 and 114 days in anti-survivin T cell anergy and activity groups, respectively. The difference was not considered significant. **(Panel B)** for patients with normal baseline IL-8, median overall survivals were 104 and 114 days in anti-survivin T cell anergy and activity groups, respectively (p=0.052). **(Panel C)** Pre-treatment NLR and IL-8 values were used to stratify patients into different groups. Overall survival was significantly increased in patients with low IL-8 and low NLR (p<0.001).

We evaluated the effects of baseline IL-8 in the context of anti-tumor T cell activity, as measured by anti-survivin ELISPOT performed on peripheral blood mononuclear cells, and pre-treatment neutrophil-to-lymphocyte ratio. In this analysis, we studied the correlation of anti-tumor T cell activity with overall survival separately for normal and high IL-8 patient groups (Figure 4A–4B). For patients with high baseline IL-8, T cell activity did not seem to associate with frequent long-term survival, whereas in patients with normal IL-8 T cell activity, effects on survival seemed more prominent. The difference was, however, not significant in this small patient group (n=23, p=0.052).

We have previously found that neutrophil-to-lymphocyte ratio (NLR) significantly predicts survival in patients treated with oncolytic adenovirus [6]. In order to verify that IL-8 is not merely reflecting the neutrophil/lymphocyte balance in these patients, we combined baseline IL-8 and neutrophil-to-lymphocyte ratio status in a survival analysis (Figure 4C). Here we found a significant improvement in the prognostic value of both factors when used in a combination approach (p<0.001).

### IL-8 blockade can augment the anti-tumor activity of oncolytic adenovirus in human ovarian tumor cell suspensions

Having established that baseline IL-8 and IL-8 change during treatment are important with regard to survival of patients treated with oncolytic adenoviruses, we next wanted to study whether this was a passive phenomenon or if there was some causal relationship. Animal studies were not possible since mice lack IL-8 [31]. Thus, we obtained ovarian tumor specimens fresh from the operating room. Importantly, these samples contained not only the tumor cells, but also cells of the tumor microenvironment, including tumor-infiltrating lymphocytes and neutrophils.

**Figure 5:**
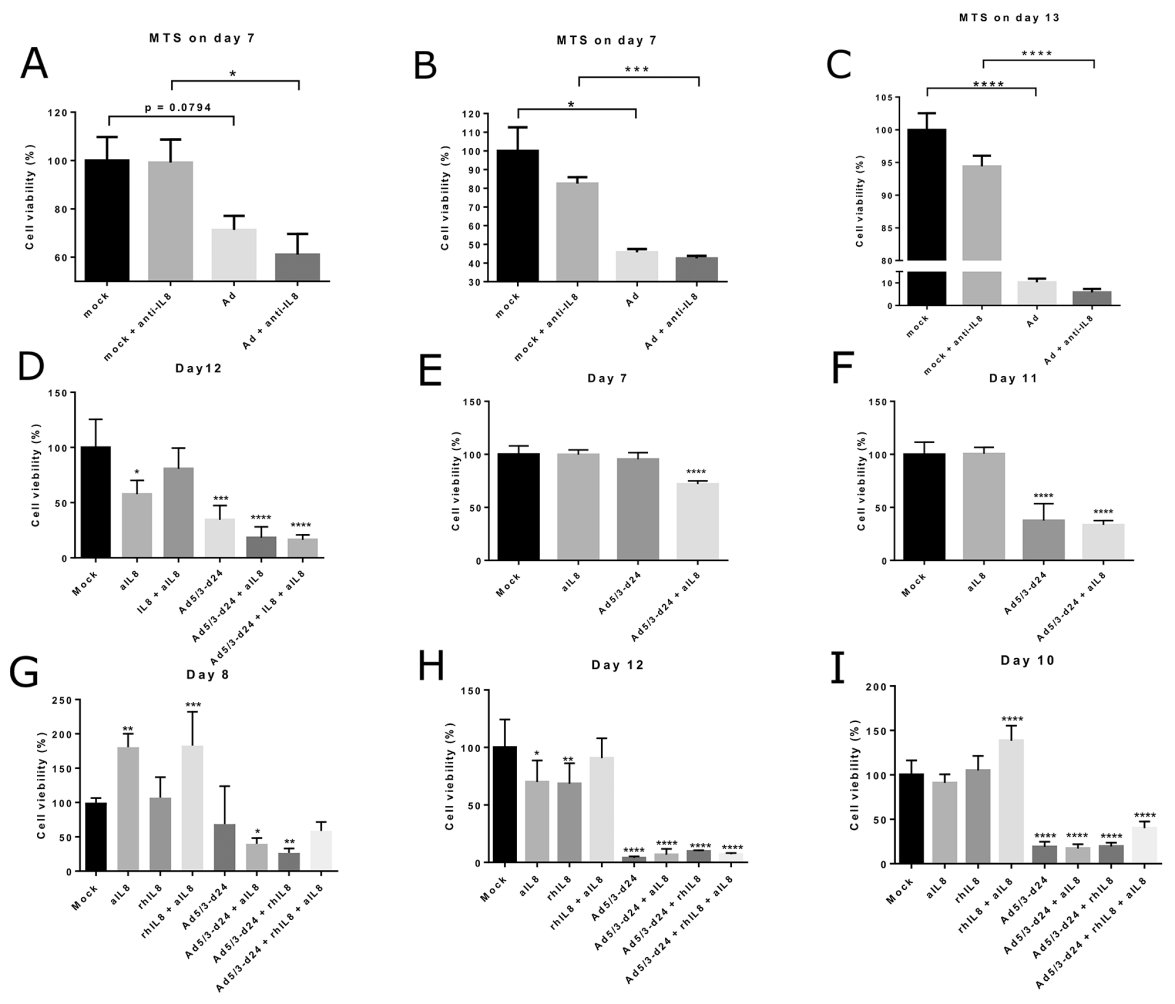
Tumor cell viability in human ovarian tumor cell suspensions following treatment with oncolytic adenovirus, recombinant IL-8 or anti-IL-8 antibody Cell viability was measured using a MTS assay on days 7-13 after the start of incubation. Viability is presented as percentage of mock group cell viability. Ad = Ad5/3-d24. aIL8=anti-IL-8=anti-IL-8 neutralizing antibody. rIL8=IL-8 = recombinant IL-8. Asterisks indicate the significance of findings: ^*^ (p<0.05), ^**^ (p<0.01), ^***^ (p<0.001), ^****^ (p<0.0001). **(Panel A)** Results from Tumor 1. **(Panels B-C)** Results from Tumor 2. **(Panel D)** Results from Tumor 4. **(Panels E-F)** Results from Tumor 5. **(Panels G-H)** Results from Tumor 6. **(Panel I)** Results from Tumor 7.

To study the feasibility of the combination of oncolytic adenovirus and IL-8 blocking antibody, we tested the cell killing abilities of the combination in an MTS cell viability assay using single cell suspensions from human ovarian tumors samples (Figure 5). In these pilot experiments, anti-IL-8 antibody increased tumor cell killing in some – but not all – conditions, which likely reflects heterogeneity between different tumors. Future studies with larger sample sizes could provide more accuracy on the magnitude of the effect. In these studies it is vital to characterize the tumors not only based on histological type, but also on the immunological and genetic bases, in order to gain more knowledge about the genetic and immunological factors that determine responses to this treatment. Of note, the effect of IL-8 blocking would be expected to be most prominent *in vivo* where the immunological effects of IL-8 play a role. Therefore, it was important to establish that anti-IL-8 antibody did not blunt the oncolytic functionality of Ad5/3-d24, which is an unarmed oncolytic adenovirus [32].

### IL-8 blockade together with adenovirus can influence TIL proliferation and activation when co-cultured with TANs isolated from ovarian tumors

**Figure 6:**
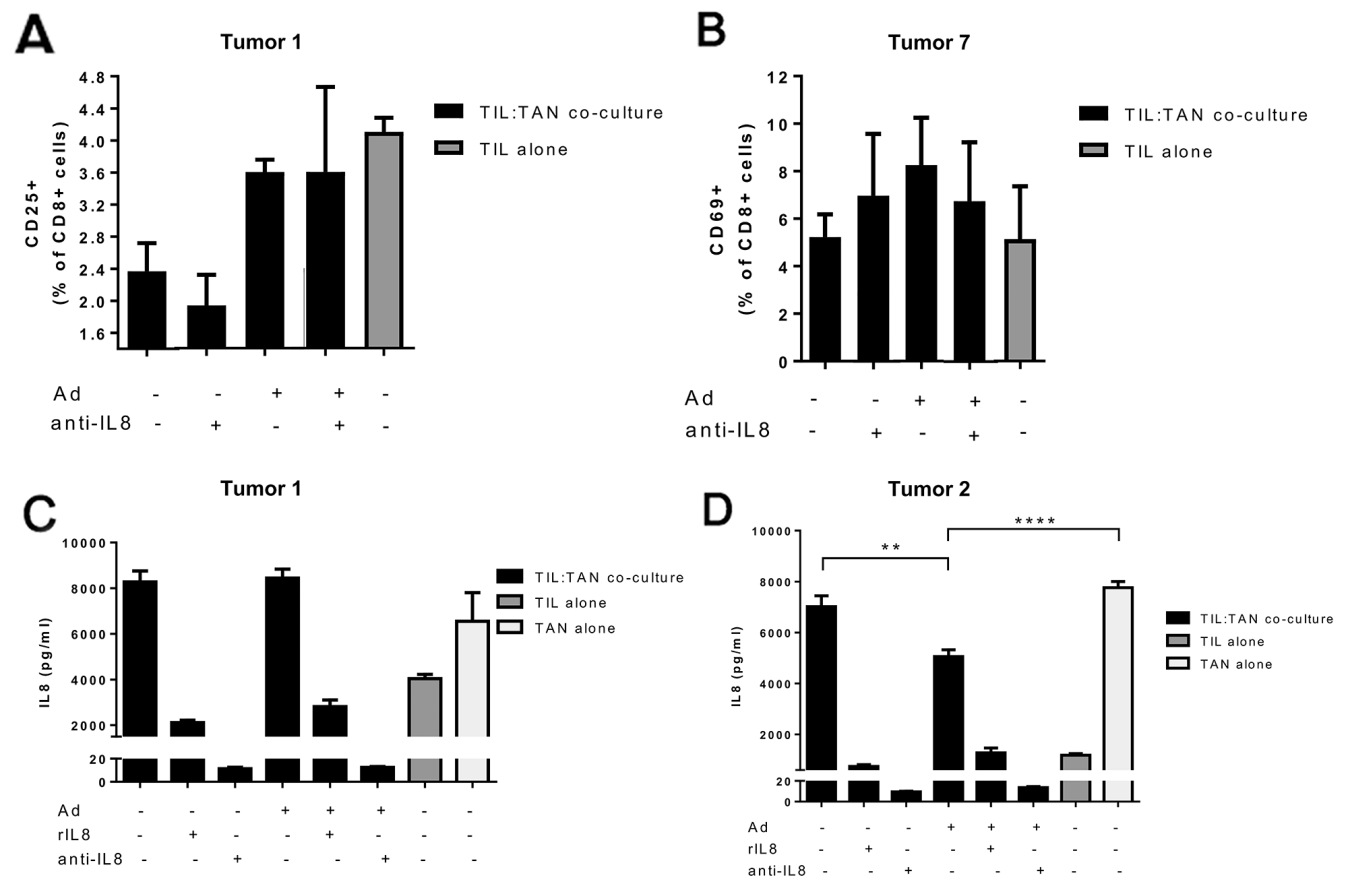
Cytotoxic T cell activation and IL-8 production in ovarian tumor derived TIL and TAN co-cultures obtained from human patients **(Panels A-B)** Cytotoxic T cell activation was measured after a 6 day incubation of TIL-TAN co-cultures or TILs alone. Bars represent the percentage of activated CD25/CD69-positive cytotoxic T cells in the culture. Ad = Ad5/3-d24. Representative examples from Tumors 1 and 7, complete data in Supplementary Figure 5. Activation marker CD25 was used for samples from Tumor 1 and activation marker CD69 for samples from Tumor 7. **(Panels C-D)** IL-8 concentration was measured after a 24 hour incubation from TIL-TAN co-cultures or TILs/TANs alone. Ad = Ad5/3-d24. rIL8 = recombinant IL-8. Asterisks indicate the significance of findings: ^*^ (p<0.05), ^**^ (p<0.01), ^***^ (p<0.001), ^****^ (p<0.0001).

Human ovarian tumor samples were processed to extract tumor infiltrating lymphocytes (TIL) and tumor associated neutrophils (TAN) for analyses of the immune effects of adenovirus and anti-IL-8 antibody combination. Of note, co-incubation of TILs with TANs did not notably suppress T cell proliferation, possible due to the strong exogenous stimuli by anti-CD3/anti-CD28 beads (Supplementary Figure 4). After a 6-day incubation, monotherapy with IL-8 blocking antibody was unable to increase T cell proliferation and in some cases even seemed to reduce the number of TILs. In contrast, addition of adenovirus into the anti-IL-8 therapy regimen was able to restore the T cell levels to same as in the mock group. A similar pattern was observed in cytotoxic T cell (CTL) activation (Figure 6A–6B, Supplementary Figure 5), but not in helper T cell activation (Supplementary Figure 6). However, it needs to be noted that the exact biological consequences of changes of this magnitude have not been clearly established, and additional functional studies would be needed to define the actual immunostimulatory impact.

In addition to TIL proliferation and activity, endogenous secretion of IL-8 was measured from TIL and/or TAN cultures after 24 h incubation *ex vivo* (Figure 6C–6D). Interestingly, TANs produced higher levels of IL-8 compared to TILs, possibly reflecting the actual situation in human tumors *in vivo* [33]. In addition, the functionality of anti-IL-8 antibody was confirmed as it was able to efficiently bind IL-8 from the suspension, leading to decrease in detected IL-8. Importantly, addition of adenovirus into the TIL:TAN co-culture did not elevate the levels of IL-8 (Figure 6C). Instead, presence of adenovirus resulted in a reduced IL-8 concentration when compared to mock treated co-cultures or TANs alone (Figure 6D). This suggests that oncolytic Ad5/3-d24 adenovirus is not likely to cause counterproductive IL-8 induction when used together with IL-8-blocking antibodies.

## DISCUSSION

In this study we initially evaluated the predictive and prognostic role of interleukin 8 in the context of adenoviral immunotherapy. We found both normal pre-treatment IL-8 and pre-post decrease to be independently associated with significantly improved overall survival. There was also preliminary evidence of higher disease control rate in patients with IL-8 decrease, although statistical significance was not reached, which was probably influenced by patient number and, more importantly, the phenomenon of inflammatory pseudo-progression which has resulted in many false negatives in cancer immunotherapy [27, 34]. While this phenomenon is currently well established, it was not fully appreciated when these treatments were given, resulting in premature abrogation of therapy and incorrect conclusions of lack of efficacy.

In an extension cohort of patients, low baseline IL-8 RNA expression in tumor biopsy or effusion samples showed a trend for longer overall survival. In contrast to intracellular and paracrine factors [26, 35], local analysis of IL-8 expression did not seem to be more sensitive (for prognostic value) over serum analysis of the soluble IL-8 protein, which is logical given the secretory nature and systemic activity of this key cytokine. Combining pre-treatment IL-8 status with anti-tumor T cell activity data or neutrophil-to-lymphocyte ratio improved the prognostic potential of both variables. In *ex vivo* studies with ovarian tumor samples we found preliminary evidence that in some instances IL-8 blockade could potentiate oncolytic adenovirus–mediated killing of tumor cells. Moreover, adenovirus seemed to be able to counteract the reduction in the number of TILs following treatment with anti-IL-8 antibodies.

The prognostic value of blood IL-8 has been previously documented in several cancers [11, 36, 37]. IL-8 has various pro-tumor effects in the tumor microenvironment [10], which explains its effects on overall survival. However, as reported here, the prognostic and, to some extent, predictive value of post-treatment IL-8 change suggests a specific role for IL-8 in the context of adenovirus based immunotherapy. Moreover, the effects of baseline IL-8 and post-treatment change seemed to be independent of each other, both providing a non-synonymous and perhaps synergistic approach for identifying patients likely to benefit from oncolytic immunotherapy (Figure 2C). This is highlighted by the fact that all patients with low baseline and subsequent decrease in IL-8 (n=4) responded to the treatment (disease control or better). However, although our data suggest that neutrophils may play a role, the full mechanistic basis of the effect of IL-8 on efficacy of adenoviral therapy is unclear and needs to be clarified in the future. In addition, the improved survival of patients with IL-8 increase compared to the no-change group, which could indicate beneficial immunological responses [38] or immunogenic cell death [25] of tumor cells in a subset of these patients who are actually responding to the treatment, calls for further studies. These studies, however, will be challenging to perform in the laboratory, since mice lack IL-8 [31].

Since study was not based on a clinical trial, it included patients with multiple different cancer types. While this heterogeneity can be seen as a weakness, it can also be considered a strength since the patient population resembles a “real life” population of cancer patients seen by a solid tumor oncologist. Furthermore, previous work has shown that IL-8 is relevant in numerous cancer types, which gives additional rationale to the approach. As there were no significant differences in the pre-treatment IL-8 levels between different tumor types, the results may illustrate a phenomenon present across several cancer types. Alternatively, tumor specific variation in IL-8 activity could eclipse tumor type specific variation, which would call for an entirely personalized approach when selecting the treatments. Nevertheless, it needs to be emphasized that the findings have to be verified in additional studies focusing on specific tumor types which also include analysis on the immunological type of the tumors. Different treatment characteristics did not alter the distribution of IL-8 changes. This suggests that neither adenovirus design nor the concomitant treatments used affected the IL-8 pathway. Some chemotherapies, such as oxaliplatin and taxanes, have been proposed to increase IL-8 signaling [39–41]. This is potentially important when considering combination treatments with oncolytic viruses in future trials, as they might interfere with immunological effects of the virus via IL-8.

Low local expression of IL-8 mRNA showed an intriguing trend for increased survival, although the finding was not statistically significant in this limited patient sample (p=0.058). However, this finding was in accord with the survival associations of systemic levels of IL-8. Thus, it seems conceivable that the IL-8 present in blood of cancer patients is at least partly tumor-derived, although the exact cell types producing the cytokine could not be identified with the microarray assay. We saw no correlation between survival and change of IL-8 RNA expression, which could indicate that other cell types, such as T cells and neutrophils, also affect the kinetics of serum IL-8. While our data suggests that baseline IL-8 protein levels may be determined by IL-8 mRNA expression by the tumor and its microenvironment, change in systemic IL-8 levels could be more influenced by treatment effect on the immune cells secreting IL-8 [42]. For example, well known sequelae of oncolytic adenovirus replication, such as pathogen associated molecular pattern recognition danger signaling [43] and immunogenic cell death [25] could shift neutrophil differentiation towards anti-tumor N1 neutrophils, as discussed below, to attenuate IL-8 production following therapy. On the other hand, increase in IL-8 levels could indicate IL-8 release from other cell types, such as macrophages, as a response to effective immunostimulation by the adenovirus, which is why it will be key to better dissect these counteracting forces going forward. It is also possible that mRNA levels may not be directly predictive of IL-8 cytokine production, since IL-8 synthesis is regulated also after transcription [42]. Nevertheless, additional studies are needed to clarify the inherent causalities as well as the primary source and kinetics of IL-8, both at the systemic level and in the tumor micro-environment.

High neutrophil counts were associated with elevated pre-treatment IL-8 concentrations, although the difference was not significant due to high variation. The association of high IL-8 and high neutrophils is logical, since neutrophil recruitment and activation is one of the main functions of IL-8 [44]. We have previously found that neutrophil-to-lymphocyte ratio predicts survival in adenovirus treated cancer patients [6]. Therefore, it was interesting that IL-8 status was able to improve its predictive ability significantly. One possible hypothesis explaining this effect is that IL-8 reflects (or even influences) neutrophil subtype along the pro-tumor/anti-tumor axis, although such a role has been previously assigned primarily to TGF-β and IFN-β [45, 46]. Again, IL-8 may have been discriminated against, since it cannot be studied in mice. Pro-tumor neutrophils have been shown to attenuate T cell responses [21, 45], in contrast to normal neutrophils that promote T cell activity [47], lending support to the notion that factors such as IL-8, which are able to influence neutrophil behavior, could have central roles in immuno-oncology and its modulation. Moreover, the ELISPOT data reported here suggests that IL-8 could influence T cell activity, possibly via immunosuppressive tumor-associated neutrophils.

Baseline blood HMGB1 (High mobility group box 1) has previously been shown to be an independent predictive and prognostic factor in patients treated with oncolytic adenovirus [26]. Thus it is intriguing that HMGB1 has been linked to the IL-8 pathway in an immunologically relevant manner [48] in the context of cancer [49]. A recent study indicated benefits for combining both HMGB1 and IL-8 inhibition in experimental gastric cancer models [50]. Based on our data, similar combinations could yield increased anti-tumor effects also in the context of adenoviral immunotherapy.

In addition to HMGB1, we have found that the expression of its receptor TIM-3 in pre-treatment tumor samples is associated with worse prognosis in adenovirus treated patients [24]. Previous studies on the function of TIM-3 in the context of cystic fibrosis have demonstrated first that TIM-3 activation induces IL-8 production [51], but also that IL-8 promotes expression of TIM-3 [52]. Thus, although the exact interdependencies of IL-8 and TIM-3 are unclear, they may relate to phenomena important for the efficacy of oncolytic adenovirus specifically and anti-tumor immunotherapy more generally. These links are also interesting considering the development of immunotherapies targeting TIM-3 [53, 54].

One important use of IL-8 quantitation, with or without concomitant neutrophil-to-lymphocyte ratio measurement, could relate to its value as a biomarker. Baseline measurement (Figure 1) would be fast, inexpensive and the test is routinely available. Moreover, given its rapid dynamics (Supplementary Figure 2), the measurement could be repeated a week after treatment initiation to identify benefiting patients. Given the high cost of new cancer drugs, in particular when used in combination, biomarkers could be useful in justifying their use, and in increasing the proportion of patients benefiting from the investment. In a patient with high baseline IL-8 and lack of IL-8 decrease, perhaps switching to another therapy is more appropriate that continuing with oncolytic immunotherapy. In our patient series, there was only a 25% chance of disease control in such patients. These sort of hypotheses should be justified in prospective trials, of course.

Perhaps the more important or interesting question is whether IL-8 can be manipulated to enhance the anti-tumor potential of adenovirus treatments. Past efforts to target IL-8 in cancer treatment have displayed promising results in preclinical models [12, 13], but clinical trials with these agents have not been completed. One approach utilized replication-incompetent oncolytic adenoviruses expressing short hairpin RNAs against IL-8 [55]. This treatment was shown to inhibit tumor progression and metastases in xenograft models. In addition to cancer, IL-8 blocking antibodies have been studied in the context of other diseases. Although efficacy trials have not yet been completed, the available early data suggests a favorable safety profile in the context of COPD and palmoplantar pustulosis [14, 15], which might predict tolerability also in cancer trials.

One caveat of previous IL-8 targeting treatments has been focus on just tumor growth inhibition and anti-angiogenesis, instead of taking into account immunological aspects. Thus, new studies of IL-8 blockade in the context of immunotherapy, and especially with oncolytic adenoviruses, are needed, but - as mentioned – these studied are complicated due to the fact that mice do not have IL-8. Xenograft studies thus only take into account human IL-8 produced by the tumor cells. Neutrophils, which were implicated in our analyses with human substrates, would probably not react to human IL-8 in xenograft studies as they would originate from the mouse. Therefore, future studies should be focused on human-derived tumor samples, although these can be sometimes difficult to obtain in large numbers, and especially TANs in order to accurately dissect these immunological phenomena. In the current flux of emerging immunotherapeutic approaches, IL-8-targeting represents an interesting example, as it has been considered as a potential treatment quite recently, but unfortunately discarded without assessing its immunological properties.

To evaluate the feasibility of combination treatment, we investigated the oncolytic and immunostimulatory effects of IL-8 blockade together with oncolytic adenovirus in human ovarian tumor samples *ex vivo*. As expected, anti-IL-8 treatment alone was not able to increase cell killing in this setting, due to lack of direct effects on tumor cells. Nevertheless, IL-8 blockade did not have harmful impacts on the oncolytic activity of adenovirus either, which is important considering a combination approach. In some cases, the combination was even able to increase tumor cell killing. Importantly, combining adenovirus to anti-IL-8 seemed to improve T cell proliferation and CTL activation, which were reduced when only anti-IL-8 antibody was present, although it needs to be noted that the exact biological significance of the change of this magnitude is not known. Of note, despite what has been proposed for some wild type viruses [17–20], we found that infection with oncolytic adenovirus did not increase IL-8 secretion. Instead, reduction of IL-8 was seen in comparison to untreated TIL-TAN co-cultures.

In summary, we have demonstrated an independent correlation between overall survival and both baseline IL-8 and post treatment IL-8 change in patients treated with adenoviral immunotherapy. Both low baseline IL-8 and IL-8 decrease were associated with superior clinical outcomes. Taking into account both variables seemed to increase the sensitivity of detection of patients likely to benefit. In contrast, prognosis was poor in patients with high IL-8 expression in their tumor or in the systemic circulation. Activity of IL-8 is likely to be at least partly mediated by the circulating and tumor-associated neutrophils. Initial studies on combination treatment with oncolytic adenovirus and IL-8 blockade in human ovarian tumor samples validated the feasibility of the combination, and provide rationale for further studies. Based on our results, IL-8 is a promising candidate biomarker for adenoviral immunotherapy and interesting target for improving the efficacy of oncolytic adenovirus. The obvious next step is to construct an oncolytic adenovirus coding for an anti-IL-8 molecule and then devise a way to test it preclinically, prior to possible clinical application.

## MATERIALS AND METHODS

### Patients treated with oncolytic adenoviruses

All patients who were treated with oncolytic adenoviruses participated in the Advanced Therapy Access Program (ATAP), which was a personalized therapy program [56]. Patients who were treated in ATAP had solid tumors refractory to standard treatments and no major organ dysfunctions. A more detailed description of the exclusion criteria has been previously reported [57]. Written informed consent was received from all of the patients before participation in the treatment program, and the studies performed on patient materials were positively evaluated by the Helsinki University Central Hospital Operative Ethics Committee (HUS 62/13/03/02/2013).

### Oncolytic viruses

Viruses that were used in the treatments have been previously published [28, 29, 58–62]. All of the analyses concerned only the first treatments that patients received with oncolytic adenoviruses. Viruses were based on either Ad5, Ad3 or a modified Ad5/3 capsid, where the Ad5 knob had been switched to Ad3 knob [29]. Some of the viruses were armed with transgene coding for GM-CSF or CD40 ligand [28, 62].

### Treatments and response evaluation

Imaging response before and after (typically at 3 months) virus treatments evaluated by computer tomography (CT) or positron emission tomography with CT (F18-FDG-PET-CT). Modified RECIST 1.1 criteria [63] were used for assessment of CT results, and previously described PET criteria [34] were used for the PET-CT imaging results. Responses were graded as progressive disease or progressive metabolic disease (PD/PMD), stable disease or stable metabolic disease (SD/SMD), minor response or minor metabolic response (MR/MMR) and complete response or complete metabolic response (CR/CMR).

### Serum IL-8 quantification

Serum IL-8 was analyzed from venous blood samples after collection using standard laboratory techniques. The laboratory reference “normal” value of 62 ng/l was used as the cutoff to determine high and low baseline IL-8 levels. The IL-8 change status was assigned based on the changes in IL-8 in samples taken during 100 days after treatment with oncolytic adenovirus by comparing post-treatment values with baseline IL-8 levels. A decrease of at least 50% was required for decrease status and an increase of at least 100% was required for increase status. If no decrease or increase was observed, the patient was assigned to the “no change” group.

### Tumor load and peripheral blood cell counts

Tumor load was assessed from pre-treatment CT and PET-CT images. Based on the metastases in different organs and size of the primary tumor a tumor load score (0-21) describing the overall tumor load was calculated according to previously described methodology [6]. In this study tumor load score was available for 60 patients. The median of the total tumor load (5) score was determined as the cutoff value for high tumor load.

Peripheral blood cell counts were obtained in the laboratory of the treating hospital using standard protocols. Baseline blood samples were obtained from patients on the day of the treatment or one day before. Neutrophil count was obtained by subtracting the lymphocyte count from total leucocyte count. Neutrophil to lymphocyte ratio was determined by dividing the baseline neutrophil count by the lymphocyte count.

### RNA microarrays

Gene expression in pre- and post-treatment tumor and liquid biopsy samples was analyzed using RNA microarrays and following computational methods as previously described [24]. Expression data was normalized using sample specific normalization to account for differential gene expression in different sample types. Baseline measurements of serum IL-8 were not available for patients with RNA microarray data. The log2 expression values were compared at baseline to determine high and low baseline gene expression. Change in the expression value between pre- and post-treatment samples was calculated and patients with negative a change were grouped into decrease group whereas patients with a positive change were assigned to increase group.

### Enzyme-linked immunospot (ELISPOT) assay

ELISPOT analysis was carried out using patient derived peripheral blood mononuclear cells (PBMCs) as described earlier [28]. Stimulation of the PBMCs was done using the tumor-associated BIRC5 PONAB peptide Survivin (ProImmune) to assess responses for a tumor-associated antigen. A total of 10 spot forming units were regarded as the lower limit of detection for the baseline and difference between pre- and post-treatment samples. ELISPOT readout changes between -3 and +3 spot forming units per well were labeled as “no change”, while less than -3 was considered decrease and above 3 increase in anti-survivin ELISPOT [25].

### Preparation of single-cell suspension from primary tumor tissue

Samples were obtained from 6 patients with ovarian tumors who had undergone surgical resection at the Helsinki University Central Hospital (Helsinki, Finland). Patients were surgically treated for expected ovarian cancer, but tumor histologies were diverse, and some tumors were considered benign in pathologist’s assessment (Supplementary Table 4). The local ethics committee positively evaluated the collection of samples, and the patients gave a written informed consent before sample collection. After collection, the samples were stored in growth medium on ice for transport. After the tumor was surgically removed, necrotic areas were removed with scissors and the remaining tumor tissue was split into small fragments (∼50 mm^3^ each) using a scalpel. For enzymatic digestion, tumor fragments were incubated in a sealed, 50 mL Falcon tube with a final volume of 25 ml enzyme solution containing serum-free RPMI 1640 supplemented with collagenase type I (170 mg/l), collagenase type IV (170 mg/l), DNase I (25 mg/ml) and elastase (25 mg/ml) (all enzymes from Worthington Biochemical). After overnight digestion at +37 °C (with rocking), which has been shown to not affect the viability of the cells [64–66] and was discovered to be necessary for producing a quality single-cell suspension, the cells were collected, passed through a 100 μm strainer and treated with ACK lysis buffer (Life Technologies, Carlsbad, CA) to eliminate red blood cells.

### Cytotoxicity assay

3.5×10^5^ cells from the single-cell suspension were plated on a 96-well plate and treated with Ad5/3-D24 (10 VP per cell) and/or neutralizing anti-IL-8 antibody (2 μg/ml; R&D Systems). Cell viability was determined 7-13 days later with CellTiter 96 AQueous One Solution Proliferation Assay (Promega, Fitchburg, WI).

### Immune cell isolation and TIL-TAN co-culture

After single-cell suspension was obtained by enzymatic digestion, tumor-infiltrating immune cells were isolated using positive selection of CD3+ (TILs) or CD15+ (TANs) with magnetic microbeads and LS columns according to manufacturer’s instructions (Miltenyi Biotec Inc, Bergisch Gladbach, Germany). For the co-culture experiment, CD3+ TILs and CD15+ TANs were plated at 1:1 ratio (2×10^5^ cells each) in a 96-well U-bottom plate containing RPMI 1640 supplemented with 10 % FBS, 20 mM L-Glutamine, 1× Pen/Strep solution, 15 mM HEPES, 50 μM 2-mercaptoethanol, 1 mM Na pyruvate and 8×10^5^ beads/ml anti-CD3/anti-CD28 Dynabeads (Thermo Fisher Scientific, Waltham, MA). In addition, recombinant human IL-8 (Peprotech, Rocky Hill, NJ) or neutralizing anti-IL-8 antibody was added to the co-culture at the final concentration of 500 ng/ml and 2 μg/ml, respectively. After 6 days, proliferation of CD3+ T cells and activation of CD4+ and CD8+ T cells was assessed by flow cytometry. Activation of the CD4+ cells was measured at this time point as both oncolytic adenoviruses and IL8 are known to affect CD4+ T cells [67–69] and 4-6 days of co-culture has been shown to be a suitable time window to analyze CD4+ T cell activity and/or function [70, 71].

### Measurement of secreted cytokines

24 hours after plating, supernatant samples were collected from the treated TIL-TAN co-cultures and stored at -80 °C until measurement. The levels of cytokines and growth factors secreted by the co-cultured TILs and TANs were measured using multiplex Flex Sets (BD Biosciences, San Jose, CA), Accuri C6 flow cytometer (BD) and FCAP Array software version 3.0.1 (BD).

### Statistical analysis

Statistical analysis was conducted using SPSS Statistics v23 (International Business Machines Corporation, Armonk, NY), Microsoft Excel (Microsoft Corporation, Redmond, WA) and GraphPad Prism (GraphPad Software, La Jolla, CA). Differences between average IL-8 levels were tested with one-way ANOVA and Student’s *t*-test. Log-rank test was utilized to compare overall survival between different IL-8 groups and subgroups defined by ELISPOT or NLR measurements. Differences in treatment responses between groups were analyzed with Fisher’s exact test. Hazard ratios for IL-8 and patient characteristics were estimated using Cox proportional hazards regression model. *P* smaller than 0.05 were regarded as statistically significant.

## SUPPLEMENTARY MATERIALS FIGURES AND TABLES




