# Supplementary material for: Interleukin 8 activity influences the efficacy of adenoviral oncolytic immunotherapy in cancer patients
Source: Oncotarget. 2018 Jan 5;9(5):6320–35. doi: 10.18632/oncotarget.23967 (PMC5814215; doi:10.18632/oncotarget.23967)
Supplement: Supplementary file 1 [file oncotarget-09-6320-s001.pdf]

Interleukin 8 activity influences the efficacy of adenoviral oncolytic immunotherapy in cancer patients

SUPPLEMENTARY MATERIALS

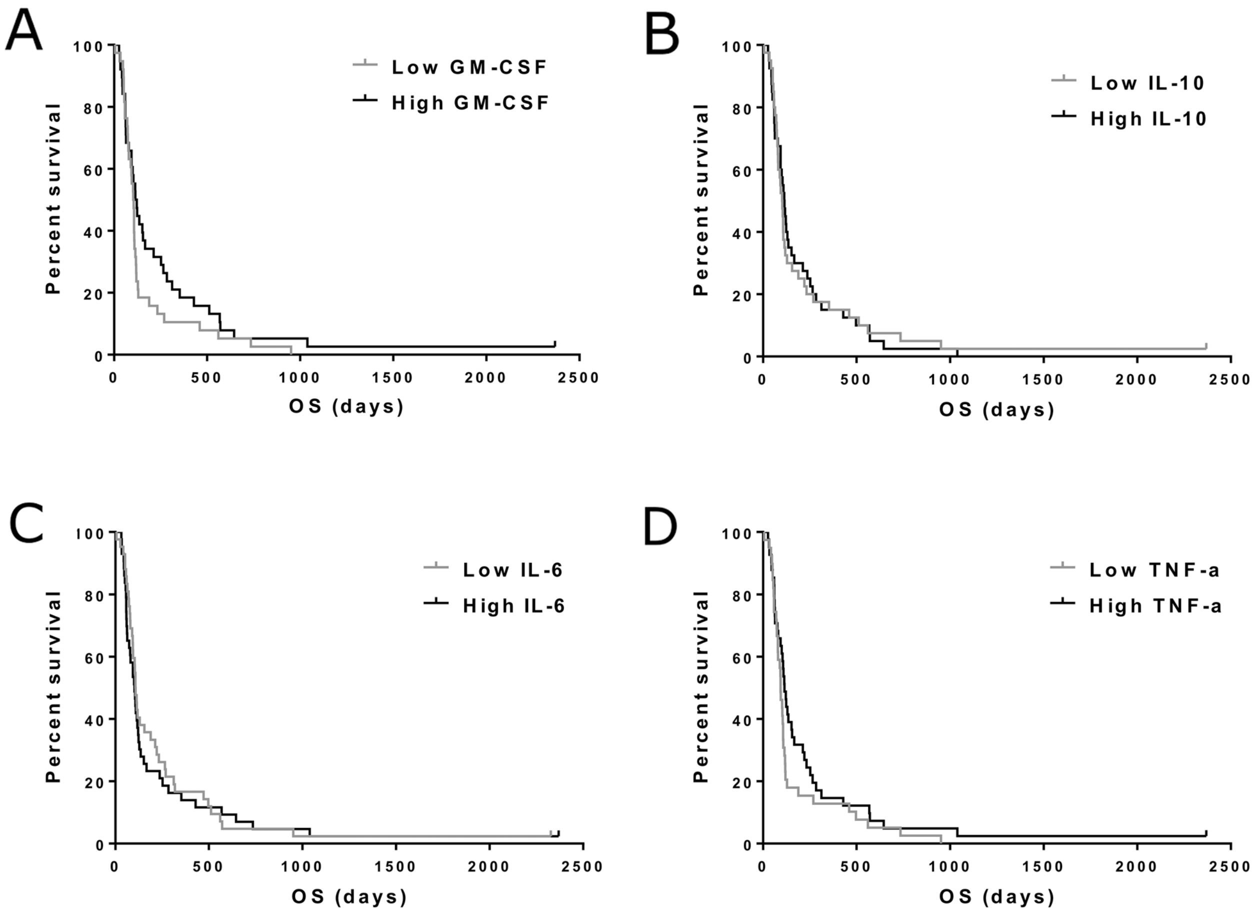

Supplementary Figure 1: Overall survival in patients in patients with low or high baseline GM-CSF, IL-10, IL-6 and TNFα. (Panels A-D): Patients were grouped to high and low baseline groups using the median baseline concentration as cutoff. No significant differences in overall survival between the groups were found.

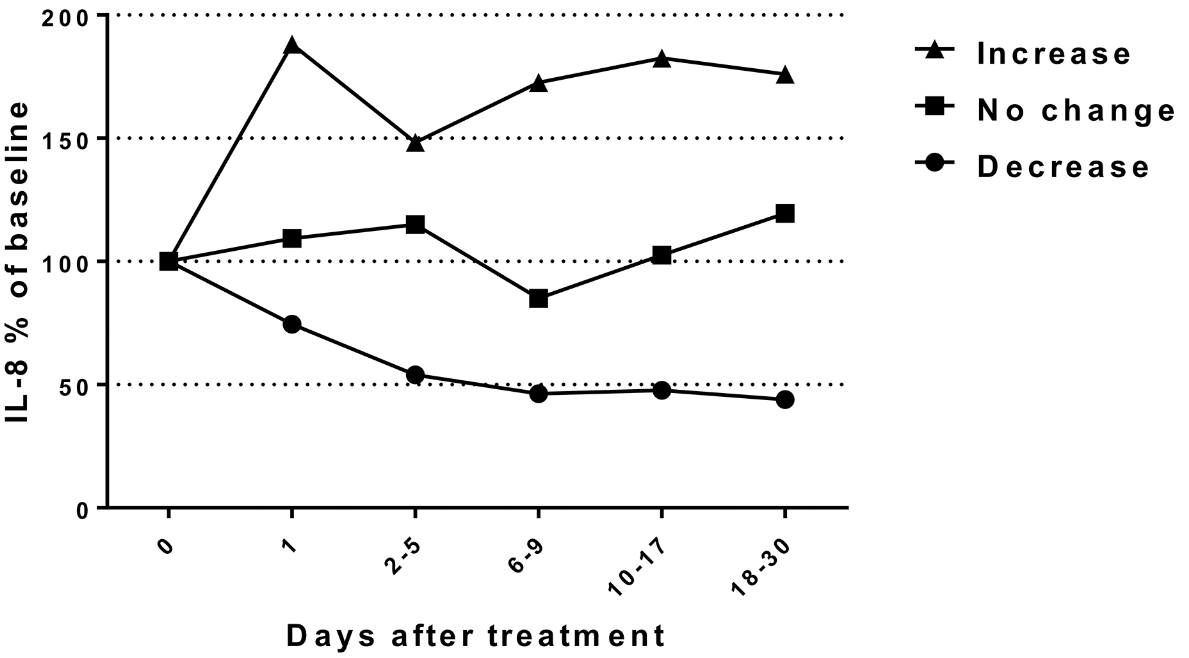

Supplementary Figure 2: Mean IL-8 concentrations 30 days after treatment in different IL-8 change groups. Patients were assigned to groups based on methodology detailed in materials and methods. Mean concentrations as percentage of the baseline value are presented for each group in different post-treatment timeframes.

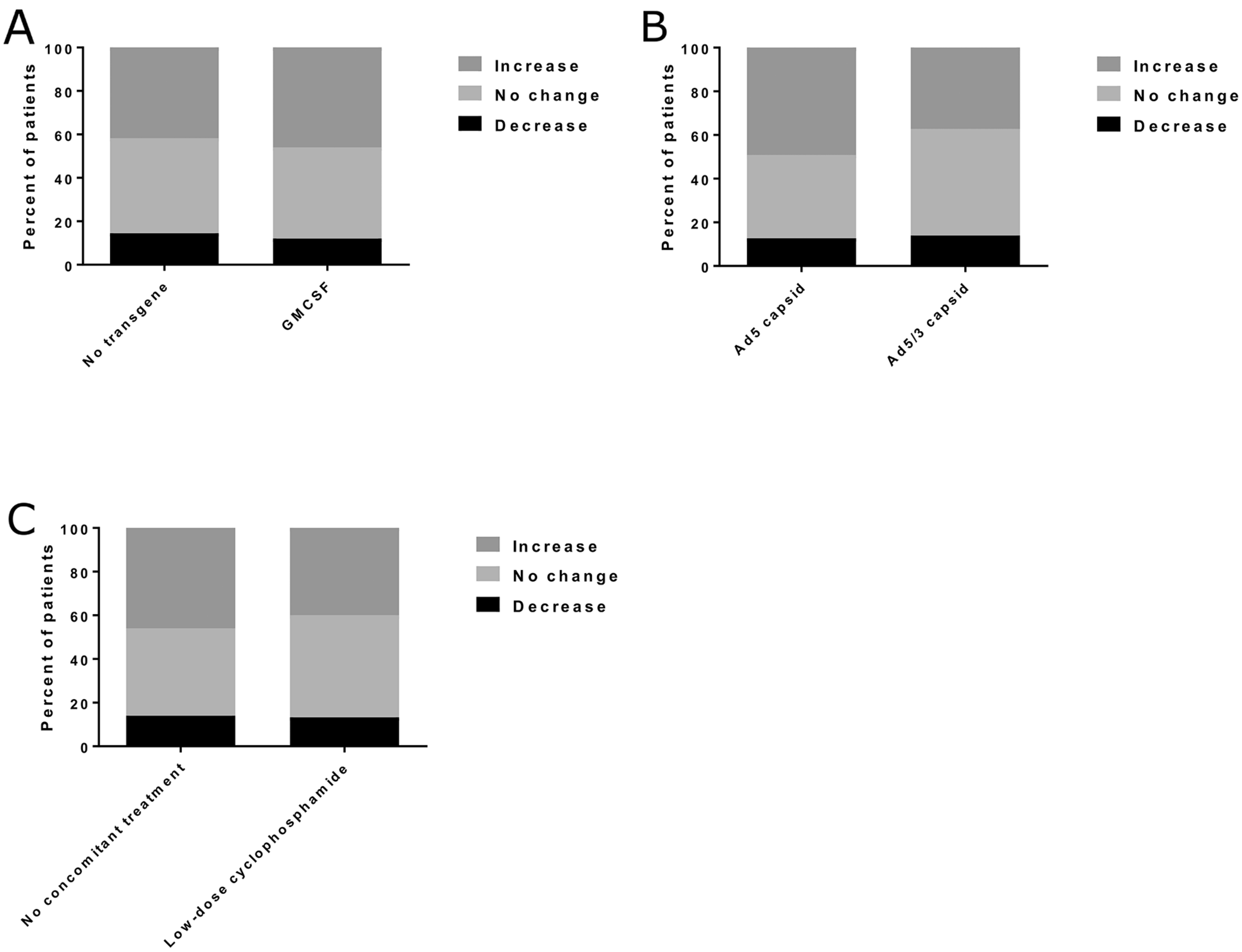

Supplementary Figure 3: Proportions of IL-8 changes in different treatment characteristics groups. **(Panels A-C)** Patients were grouped based on viral capsid, viral transgene or concomitant treatment used in the first adenovirus treatment. The differences between the groups were not considered significant.

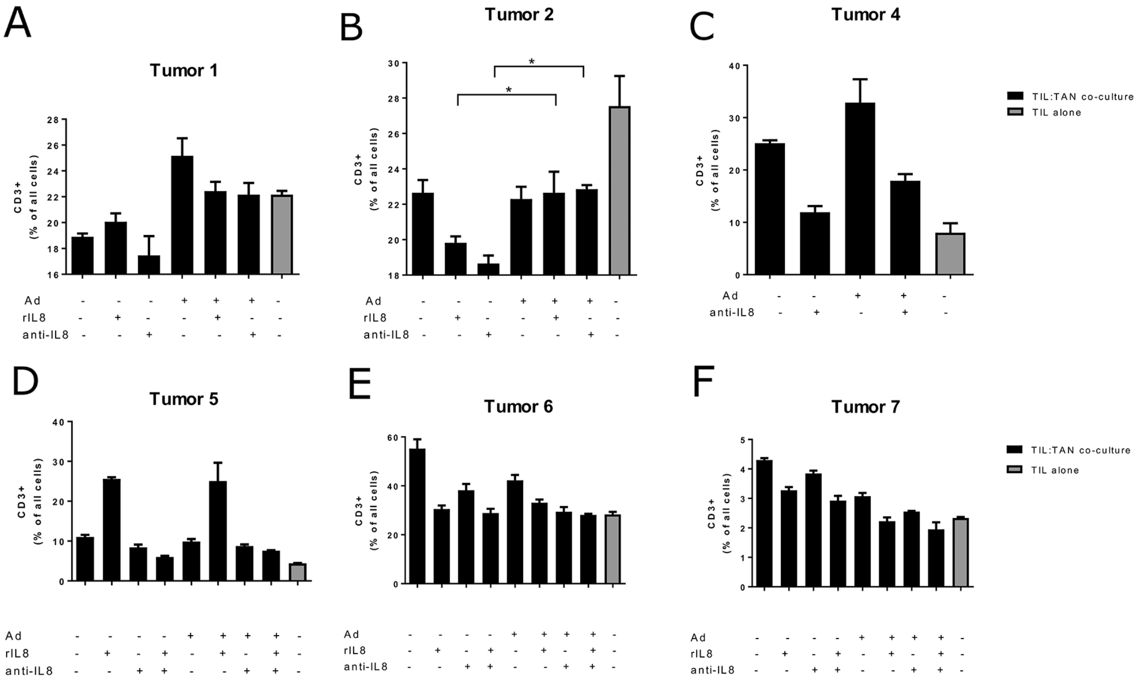

Supplementary Figure 4: T cell proliferation in ovarian tumor derived TIL and TAN co-cultures obtained from human patients. (Panels A-F) T cell proliferation was measured after 6 day incubation of TIL-TAN co-cultures or TILs alone. Bars represent the percentage of T cells in the culture. Ad = Ad5/3-d24. rIL8 = recombinant IL-8. Asterisks indicate the significance of findings: * ($p<0.05$), ** ($p<0.01$), *** ($p<0.001$), **** ($p<0.0001$).

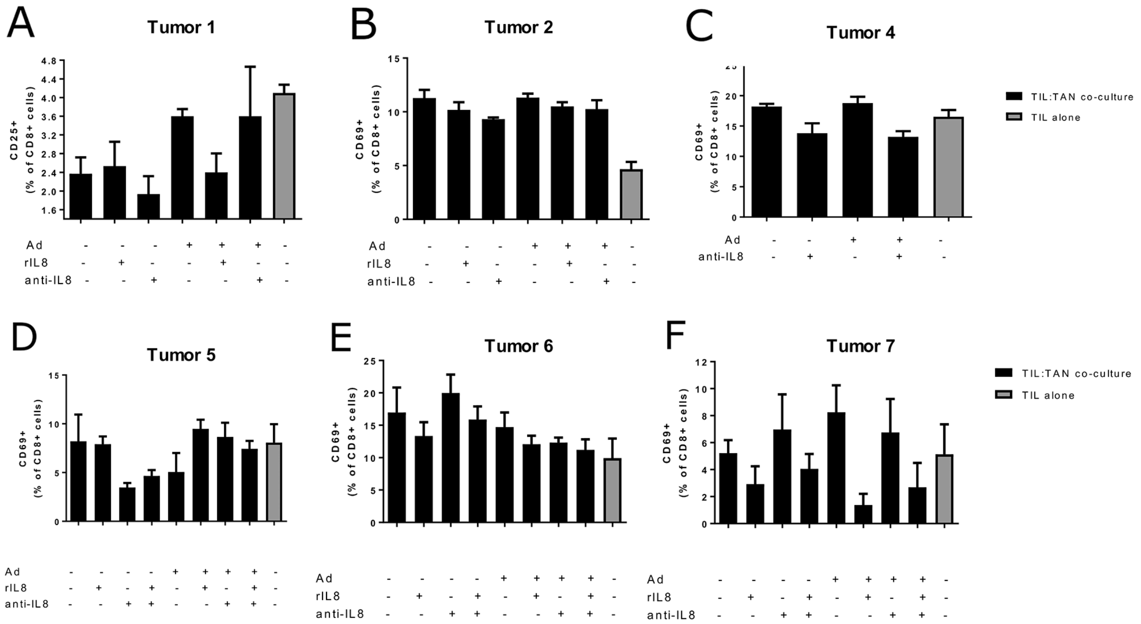

Supplementary Figure 5: Cytotoxic T cell activation in ovarian tumor derived TIL and TAN co-cultures obtained from human patients. Cytotoxic T cell activation was measured after a 6 day incubation of TIL-TAN co-cultures or TILs alone. Bars represent the percentage of activated CD25/CD69-positive cytotoxic T cells in the culture. Ad = Ad5/3-d24. rIL8 = recombinant IL-8. **(Panel A)**: Activation marker CD25 was used for samples from Tumor 1. **(Panels B-F)**: Activation marker CD69 was used for samples from other tumors.

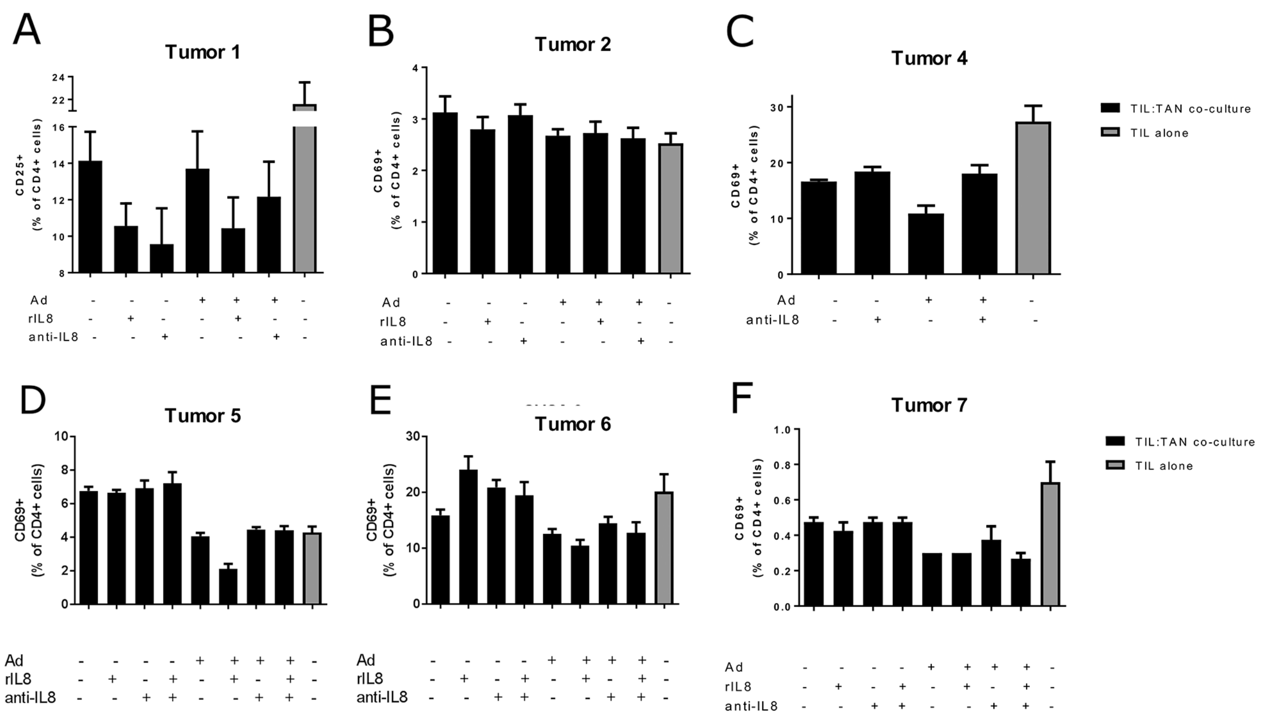

Supplementary Figure 6: Helper T cell activation in ovarian tumor derived TIL and TAN co-cultures obtained from human patients. Helper T cell activation was measured after 6 day incubation of TIL-TAN co-cultures or TILs alone. Bars represent the percentage of T cells in the culture. Ad = Ad5/3-d24. rIL8 = recombinant IL-8. **(Panel A)**: Activation marker CD25 was used for samples from Tumor 1. **(Panels B-F)**: Activation marker CD69 was used for samples from other tumors.

Supplementary Table 1: Patient characteristics.

See Supplementary File 1

Supplementary Table 2: Multivariate analysis for prognostic value of baseline IL-8 and IL-8 change

		Hazard ratio (HR) for cancer mortality (n=98)	
		**P value**	**HR (95% CI)**
Age		0.395	0.992 (0.974-1.010)
Sex (female/male)		0.804	1.073 (0.616-1.868)
Tumor type		0.360	
(vs. Panc/Chol/HCC)	CRC/Gastric	0.521	1.269 (0.612-2.631)
	Melanoma	0.372	0.614 (0.210-1.793)
	Lung	0.819	1.140 (0.371-3.503)
	Gynecological	0.241	0.620 (0.278-1.379)
	Other	0.286	0.674 (0.327-1.390)
WHO (low/high)		0.002	0.467 (0.288-0.755)
Baseline IL-8 (low/high)		0.010	0.502 (0.297-0.847)
IL-8 change		0.004	
(vs. No change)	Increase	0.060	0.576 (0.324-1.024)
	Decrease	0.001	0.270 (0.122-0.596)

Supplementary Table 3: Patients included in tumor gene expression analyses

Patient ID	Age	Sex[a]	WHO score	Diagnosis	Sample[b]	Biopsied tumor	Virus	Imaging response[c]	OS days
C341	66	F	1	Colorectal cancer	B	Liver	Ad5/3-hTERT-CD40L	SMD	314
I398	25	F	1	Melanoma	B	Lymph node	Ad5/3-E2F-d24-GMCSF	PMD	127
O279	62	F	2	Ovarian cancer	B	Liver	Ad5/3-d24-GMCSF	SMD	101
O340	75	F	0	Ovarian cancer	B	Left rib	Ad5/3-E2F-d24-GMCSF	MMR	444
O38	36	F	1	Ovarian cancer	B	Breast	Ad5/3-d24-GMCSF	MMR	336
P407	71	M	2	Prostate cancer	B	Prostate	Ad5-d24-RGD-GMCSF	PMD	100
R356	40	F	1	Breast cancer	B	Right Breast	Ad5/3-E2F-d24-GMCSF	PMD	102
R367	59	F	1	Breast cancer	B	Sternum	Ad3-hTERT-E1A	MMR	204
X373	57	F	2	Cervical cancer	B	Kidney	Ad5/3-E2F-d24-GMCSF	SMD	144
H333	61	F	2	Pancreatic cancer	A	Abdomen	Ad5/3-E2F-d24-GMCSF	N/A	50
H339	65	M	1	Pancreatic cancer	A	Abdomen	Ad5/3-E2F-d24-GMCSF	N/A	29
O391	53	F	2	Ovarian cancer	A	Abdomen	Ad5/3-E2F-d24-GMCSF	PMD	143
C335	71	F	2	Colorectal cancer	P	Pleura	Ad5/3-hTERT-CD40L	N/A	34
K326	65	M	2	Lung cancer	P	Pleura	Ad3-hTERT-E1A	N/A	72
M329	67	M	3	Mesothelioma	P	Pleura	Ad5/3-hTERT-CD40L	N/A	64

[a] F=Female; M=Male.
[b] A= Ascites fluid; B=Biopsy; P=Pleural fluid.
[c] MMR=Minor metabolic response; SMD=Stable metabolic disease; PMD=Progressive metabolic disease; N/A=Not available.

Supplementary Table 4: Ovarian tumor patients and tumor histology types

Tumor number	Histological diagnosis
Tumor 1	Mucinous cystadenoma
Tumor 2	Clear cell carcinoma stage IC1
Tumor 4	Semimalignant serous cystadenoma
Tumor 5	High grade serous carcinoma stage IIIC
Tumor 6	Benign fibroma
Tumor 7	Mucinous carcinoma stage IC2
