# Supplementary material for: Interleukin 8 activity influences the efficacy of adenoviral oncolytic immunotherapy in cancer patients
Source: Oncotarget. 2018 Jan 5;9(5):6320–35. doi: 10.18632/oncotarget.23967 (PMC5814215; doi:10.18632/oncotarget.23967)
Supplement: Supplementary file 2 [file oncotarget-09-6320-s002.docx]

**Supplementary Table 1: Patient characteristics**

|  |  |
| --- | --- |
| *Sex, no. of patients (total N=103)* |  |
| Male | 45 |
| Female | 58 |
|  |  |
| *Age (years)* |  |
| Median | 58 |
| Range | 5-77 |
|  |  |
| *WHO performance status (0-5), no. of patients* |  |
| 0 | 11 |
| 1 | 48 |
| 2 | 34 |
| 3 | 10 |
|  |  |
| *Tumor type, no. of patients* |  |
| Ovarian cancer | 18 |
| Colorectal cancer | 14 |
| Sarcoma | 11 |
| Pancreatic cancer | 8 |
| Prostate cancer | 7 |
| Breast cancer | 7 |
| Melanoma | 6 |
| Lung cancer | 6 |
| Head and neck cancer | 5 |
| Mesothelioma | 4 |
| Cholangiocancer | 4 |
| Gastric cancer | 3 |
| Neuroblastoma | 2 |
| Anal cancer | 1 |
| Esophageal cancer | 1 |
| Hepatocellular cancer | 1 |
| Neuroendocrine cancer | 1 |
| Thyroid cancer | 1 |
| Urinary bladder cancer | 1 |
| Endometrial cancer | 1 |
| Cervical cancer | 1 |
|  |  |
| *Previous treatments, no. of patients* |  |
| Surgery | 68 |
| Chemotherapy | 103 |
| Radiotherapy  Immunotherapy | 48  11 |
|  |  |
| *Virus used for treatment, no. of patients* |  |
| ICOVIR-7 | 23 |
| Ad5-d24-RGD | 9 |
| Ad5-d24-GMCSF | 18 |
| Ad5/3-cox2L-d24 | 18 |
| Ad5-RGD-d24-GMCSF | 7 |
| Ad5/3-d24-GMCSF | 28 |
|  |  |
| *Radiological responses, no. of patients (n=59)* |  |
| Progresssive disease | 32 |
| Stable disease | 17 |
| Minor response | 7 |
| Partial response | 1 |
| Complete response | 2 |
|  |  |
|  |  |
